# Effect of Various Types of Sugar Binder on the Physical Properties of Gum Powders Prepared via Fluidized-Bed Agglomeration

**DOI:** 10.3390/foods10061387

**Published:** 2021-06-16

**Authors:** Donghyeon Lee, Gyeongeon Min, Wooseok Roh, Byoungseung Yoo

**Affiliations:** Department of Food Science and Biotechnology, Dongguk University-Seoul, Goyang 410-820, Gyeonggi, Korea; ldhdongdong@gmail.com (D.L.); gyeongeonmin25@gmail.com (G.M.); 300seok@gmail.com (W.R.)

**Keywords:** physical property, gum powder, sugar binder, particle agglomeration

## Abstract

Particle agglomeration of fine gum powders to improve their physical and morphological characteristics is of crucial importance. Changes in the physical properties of guar gum, locust bean gum, and carboxymethyl cellulose powders subjected to fluidized-bed agglomeration with various sugar types as the binder were examined. The agglomerates with sugar binders had much larger particles (D_50_) and higher porosity (ε) than the corresponding fine gum powders, as confirmed by particle-size-distribution analysis and scanning electron microscopy. In particular, the carboxymethyl cellulose agglomerate exhibited much higher D_50_ and ε values than the original fine gum powder, with sorbitol as the binder resulting in the highest D_50_ and ε values. Except for guar gum with sorbitol as the binder, the guar gum and locust bean gum agglomerates with the other sugar binders showed lower Carr index and Hausner ratio values (thus exhibiting better flowability and lower cohesiveness) than the original powders, whereas those of the carboxymethyl cellulose agglomerates were higher. These findings indicate that the physical and structural properties of gum powders can be greatly improved according to the type of gum and sugar solution used in the agglomeration process.

## 1. Introduction

Fluidized-bed agglomeration (FBA) causes fine particles to cluster into larger ones, leading to a porous aggregate much larger in size than the original particles. The agglomerate growth mechanism of FBA consists of three steps: (1) wetting of the liquid binder over a dry powder and nucleating primary particles, (2) coalescing of nuclei and agglomerate consolidation by compaction, and (3) agglomerate break-up and attrition by agitation [1]. The FBA process improves the flowability, appearance, handling, and dispersion or dissolution of fine particles by modifying their size, shape, density, and/or porosity [2]. In the food industry, agglomeration is applied when the principal objective is to produce porous agglomerates with a suitable particle size that can be dispersed or dissolved quickly in a liquid [3]. Moreover, the agglomerated products can be final consumer foods (e.g., instant powdered drinks) or products (e.g., gum or starch as a thickening or gelling agent) that are used in food processing [4]. In particular, a wide variety of food thickening or gelling agents mainly consisting of gums that must be agglomerated to avoid the formation of lumps or undissolved sediment during hydration. However, there is not much information about using FBA on gum powders. Only a few researchers [5,6,7] have studied using FBA on xanthan gum (XG) powder mainly used as an instant thickener for patients with swallowing difficulty. They reported that the use of sugar and gum binder liquids could greatly enhance the physical properties of XG-based thickeners. Lee and Yoo [4] found that the physical properties of agglomerated galactomannans could be considerably influenced by the particle growth during agglomeration and the concentration of maltodextrin solution used as a binder. They also suggested that the investigation of galactomannan gums agglomerated with different sugar binders is needed to understand the intermolecular interaction between galactomannans and sugar binder solution.

In the FBA process, the binder solution usually undergoes a phase change due to the removal of the solvent, which leaves the solute behind as an adhesive [2]. Therefore, in FBA, both particle enlargement and drying can be carried out in the same equipment. The binder type is one of the parameters most widely varied in the FBA process to improve and modify the structure of agglomerated powders [8]. Many different types of binders have been used in particle size growth because the binder type plays a vital role in the physical and structural properties of agglomerates [9]. However, different binders at the same concentration have very different enlargement characteristics due to the intermolecular interactions between the particles in the presence of the binder solution [8]. It is well known that the different structural characteristics of agglomerates are considerably influenced by binder type [9]. Recently, several researchers [5,10,11,12,13] have described the effect of different binders on the FBA process to produce agglomerates for the food industry. Despite the importance of FBA, there is little information available on the physical effects of adding a sugar binder during gum-powder agglomeration.

In the food industry, guar gum (GG), locust bean gum (LBG), and carboxymethyl cellulose (CMC) have been commonly used as thickening agents, or for dysphagia management because they are easy to swallow [14,15,16]. However, the underlying process of how FBA works on these gum powders is limited. Moreover, no attempt has yet been made to examine the effect of different types of sugar binders on the physical and structural properties of the agglomerates. The aim of this study was to examine the effect of various sugar binder solutions on the physical properties of agglomerates prepared via FBA by comparing them with their original fine gum powder. During these evaluations, the physical and structural differences between GG, LBG, and CMC agglomerates prepared with different sugar binders were also investigated. The information presented in this study will provide additional knowledge to develop the agglomerated gum powders for thickening agents or food thickeners for people with dysphagia.

## 2. Materials and Methods

### 2.1. Materials

Commercial GG (Habgen Guargums Ltd., Karachi, Pakistan), LBG (Incom Co., Mersin, Turkey), and CMC (Bolak Co., Ltd., Incheon, Korea) were used to produce agglomerates via the FBA process. The following sugars were used to prepare binder solutions: glucose (Samyang Co., Ltd., Seongnam, Korea), sucrose (Samyang Co., Ltd., Seongnam, Korea), lactose (Meggle Co., Ltd., Wasserburg, Germany), and sorbitol (Samyang Co., Ltd., Seongnam, Korea).

### 2.2. FBA Process

A top-spray fluidized-bed granulator (Fluid Bed Lab System, Dae Ho Technology Co., Ltd., Hwaseong, Korea) was used for FBA. Binders were prepared by completely dissolving glucose, sucrose, lactose, or sorbitol in distilled water at room temperature to make a 10% (w/w) solution. Original fine gum powders (1500 g for GG and LBG; 750 g for CMC) were first placed in the product container and fluidized by an upward-flowing hot-air stream. The binder (1000 mL for GG and LBG; 500 mL for CMC) was then pumped through a peristaltic tube at a speed of 20 mL/min and sprayed into small droplets through a fluid spray nozzle onto the flowing powder with a pressure of 1.5 bar. Throughout the spraying process, the inlet air and product temperatures were adjusted to remain at 75 ± 1.0 °C and 53 ± 1.0 °C, respectively. Meanwhile, the blower and damper were also controlled to 70% and 30%, respectively. After the binder solution had been exhausted, the product was cooled and dried with fluidizing air at room temperature for 10 min.

### 2.3. Particle Size Distribution (PSD) Measurements

PSD measurements were made using a Malvern Mastersizer (Mastersizer 3000E, Malvern Instruments Ltd., Worcestershire, UK) based on the volume distribution. The D_10_, D_50_, and D_90_ values were the cumulative particle diameters where 10%, 50%, and 90% of the sample had smaller particle sizes than the given average particle size. The span index was calculated as (D_90_–D_10_)/D_50_.

### 2.4. Capillary Viscosity (η_c_) Measurements of the Sugar Solutions

To measure the η_c_ values, 6 mL of binder solution was injected into a Cannon-Fenske capillary viscometer (Cannon Instrument Co., State College, PA, USA), and then the whole viscometer was immersed in a water bath (HBS 1000, Eyela, Tokyo, Japan) for 60 min to become thermostatically equilibrated at 25 °C. Once equilibration had been completed, η_c_ was measured as suggested by Bak and Yoo [17]. All measurements were carried out in triplicate.

### 2.5. Flowability and Cohesiveness Measurements

Hausner ratio (HR) and Carr index (CI) values were calculated to measure the cohesiveness and flowability of a powder, respectively. A powder was poured into a 100 mL graduated glass cylinder, and then the cylinder was tapped 1250 times with a tap density tester (BT-301, K-ONE Ltd., Seoul, Korea). Bulk (ρ_bulk_) and tapped density (ρ_tapped_) were calculated by dividing the weight of the poured powder into the volume occupied by the powders before and after being tapped, respectively. From ρ_bulk_ and ρ_tapped_, HR and CI can be respectively calculated using the following equations:

(1) $$\mathrm{HR}=\frac{\rho_{\mathrm{tapped}}}{\rho_{\mathrm{bulk}}}$$

(2) $$\mathrm{CI}=\frac{\rho_{\mathrm{tapped}}-\rho_{\mathrm{bulk}}}{\rho_{\mathrm{tapped}}}\times 100\;(\%)$$

Cohesiveness of the powder is considered low when HR < 1.2, intermediate for 1.2 < HR < 1.4, and high for HR > 1.4 [18]. Meanwhile, the flowability of a powder is considered very good when CI < 15, good for 15 < CI < 20, fair for 20 < CI < 35, bad for 35 < CI < 45, and very bad for CI > 45 [19].

### 2.6. Particle Density (ρ_particle_) and Porosity (ε) Measurements

To calculate ρ_particle_, a powder (1.0 g) was poured into a 10 mL graduated glass cylinder, and then 5 mL of petroleum ether was added to form a suspension that filled the empty spaces within the powder. Powder stuck to the wall of the cylinder due to the pouring step, and was cleaned off with 1 mL of additional petroleum ether. After making the suspension, ρ_particle_ was calculated as:

(3) $$\rho_{\mathrm{particle}}=\frac{W_{p}}{V_{t}-6}$$

where W_p_ is the mass of the powder (g) and V_t_ is the total volume of the suspension (mL).

The porosity (ε) of the powder was then calculated as follows:

(4) $$\varepsilon =\frac{\rho_{\mathrm{particle}}-\rho_{\mathrm{tapped}}}{\rho_{\mathrm{particle}}}\times 100\;(\%).$$

### 2.7. Powder Dispersibility

This was estimated from the turbidity of the powder dispersed in water. First, 0.3 g of agglomerate was added to 100 mL of distilled water, which was immediately stirred to disperse the agglomerate. The turbidity of the dispersion was then measured via a turbidimeter (AQ4500, Thermo Fisher Scientific Inc., Waltham, MA, USA) after stirring for 10, 20, 30, 60, 90, 120, 150, and 180 s. However, the turbidities of the suspended fine gum powders and the LBG agglomerates were not measured because too much noise was caused by the formation of large lumps during dispersal.

### 2.8. Scanning Electron Microscopy (SEM)

Powders were attached to an aluminum stub using double-sided adhesive carbon tape and coated with platinum–palladium in a vacuum. Particle morphology was evaluated via SEM (Hitachi S-3000 N SEM, Hitachi Ltd., Tokyo, Japan) operating at 20 kV from images collected at 100× magnification.

### 2.9. Color Analysis

CIELab color space was used to investigate the visual characteristics of the powders, expressed as L * (lightness), a * (redness), and b * (yellowness), and measured using a color reader (CR-20, Konica Minolta, Tokyo, Japan). C * (chroma) from the a * and b * measurements was calculated as:

(5) $${C\;}^{*}=\sqrt{{\left({a\;}^{*}\right)}^{2}+{\left({b\;}^{*}\right)}^{2}}.$$

The total color difference (ΔE *) between the fine gum powder and its agglomerate was calculated in terms of ΔL * (L * − L_0_ *), Δa * (a * − a_0_ *), and Δb * (b * − b_0_ *), where L_0_ *, a_0_ *, and b_0_ * are the color values of the original fine gum powder, as follows:

(6) $$\Delta E^{*}=\sqrt{{\left(\Delta {L\;}^{*}\right)}^{2}+{\left(\Delta {a\;}^{*}\right)}^{2}+{\left(\Delta {b\;}^{*}\right)}^{2}}.$$

### 2.10. Statistical Analysis

The physical parameters of the fine gum powders and agglomerates are reported as the mean ± standard deviation. Comparisons among samples were assessed with Duncan’s multiple range tests using SAS version 9.4 (SAS Institute, Cary, NC, USA). The level of statistical significance was set at *p* < 0.05.

## 3. Results and Discussion

### 3.1. Particle Size Distribution (PSD) and Particle Diameter

**Figure 1:**
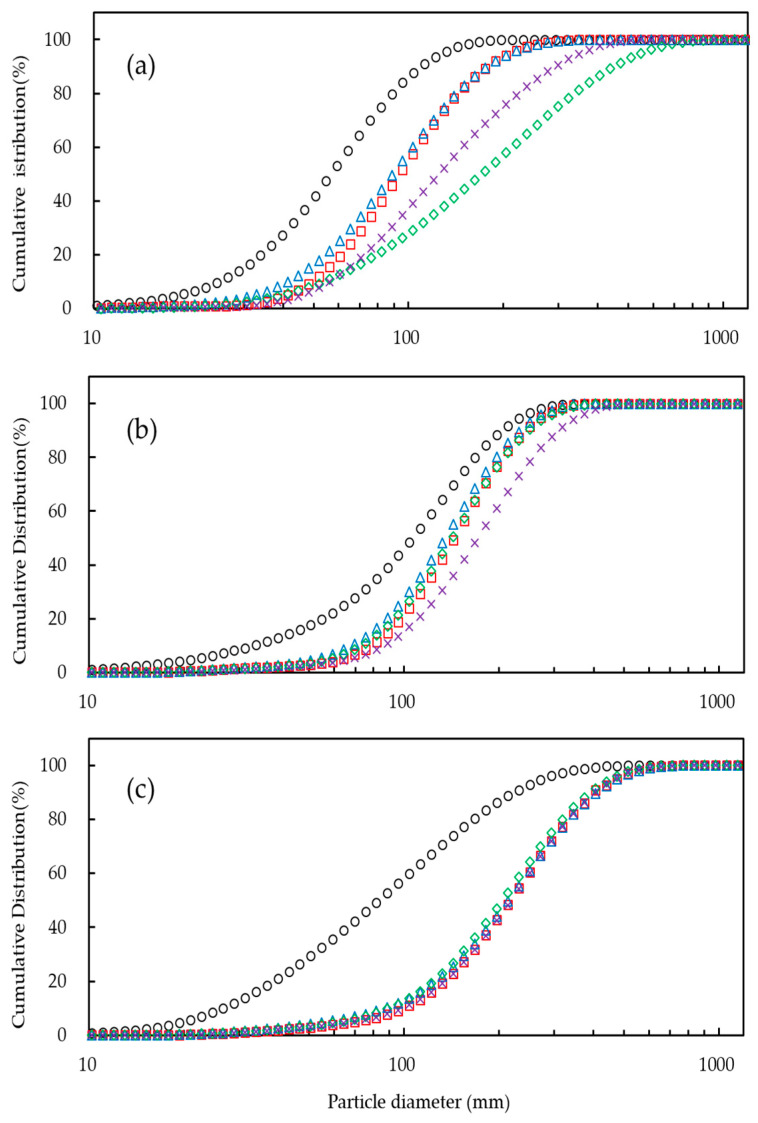
Cumulative distribution of fine gum powders (○) and gum agglomerates (**a**) GG, (**b**) LBG, and (**c**) CMC with different binders: (□) glucose, (△) sucrose, (◇) lactose, and (×) sorbitol.

Table 1 and Figure 1 present the results for the particle diameters and PSDs of the original fine gum powder and agglomerate, which were greatly affected by different sugar binders. The agglomerates had significantly larger particle sizes than the fine gum powders, indicating that the sugar addition was very effective for the enlargement of the gum particle size. The agglomerate with sugar binders increased the particle size values (D_50_) in the order of sucrose < glucose < sorbitol < lactose for GG, sucrose < glucose = lactose < sorbitol for LBG, and lactose < sucrose < glucose = sorbitol for CMC. Agglomerated GG (117–200% with lactose and sorbitol) and agglomerated CMC (151–163% with all sugars) showed the highest percentage increases in particle size compared to their original fine gum powders. In particular, among all samples, the highest percentage increase (200%) in particle size was found for the agglomerated GG powder with lactose. In general, the agglomerate with sugar binders had percentage increases in the order of LBG (27–61%) < GG (39–200%) < CMC (151–163%), thereby showing dependency on the gum type. The highest particle size of the CMC agglomerates could have resulted from the formation of strong solid bridges that linked the primary particles with the sugar binder. From these results, it was found that the sugars had different effects on particle growth depending on the type of gum. In general, it is known that high viscosity of the binder solution can occur due to the formation of larger droplets during spraying, resulting in increased particle size [20,21].

The capillary viscosity (η_c_) values of the sugar binder solutions used in this study were in the following order (low to high): sorbitol < glucose < sucrose < lactose (Table 2), indicating that there was no relationship between η_c_ and D_50_ for any of the gum powders. This was in contradiction to the results obtained by Lee and Yoo [6], who observed that there was a significant relationship between D_50_ and η_c_ of agglomerated XG powder. From these results, it can be concluded that the effect of binder viscosity on particle size growth was greatly affected by the type of gum.

The span values (1.11–1.32) of the LBG and CMC agglomerates were much lower than those (1.60–2.37) of the fine powders, showing that a narrow size distribution with homogenous particles occurred when using a sugar binder (Table 1). In contrast, the span values (1.54–2.31) of the GG agglomerates (except for the one with glucose) were much higher than that (1.48) of the fine powder. In addition, all of the agglomerates showed large variations in span value regardless of the sugar type. The higher span values could be due to the mixing of small agglomerates with large agglomerates due to the breaking up of the latter during the agglomeration process. In general, the span values of the agglomerates increased in the order of LBG (1.11–1.32) < CMC (1.38–1.50) < GG (1.39–2.31), again showing dependency on the gum type. This is probably because GG is more friable than the others due to stronger friction between the particles and between them and the product vessel caused by the high-pressure airflow [22]. In particular, among the agglomerates, the GG agglomerate with lactose with the highest span value (2.31) had the widest PSD. Thus, the span was greatly influenced by the strength of the bridge structures connecting the primary particles with the sugar binder [4,23]. According to Johansen and Schæfer [20], the agglomerates can simultaneously be broken up and grow due to both friction forces and particle coalescence in the granulator. Therefore, GG was more friable than the others due to higher friction forces between the particles and between the particles and the fluidizing vessel [22]. A similar result was also reported for agglomerated galactomannans in the presence of dextrin binder solution at different concentrations [4]. Therefore, it can be concluded that the particle size and PSD of the GG agglomerates prepared with sugar binders were considerably affected. In particular, all of the agglomerated gums prepared with glucose showed the lowest span values when compared with other sugars, making their particle sizes less diverse than the other agglomerates. In addition, their span values were also lower than the fine powders, which can be attributed to the lower friability due to more homogenous particles with more regular shapes than the agglomerates formed with the other sugars, thus using glucose was more effective at decreasing the break-up rate of the agglomerated particles. Relatedly, higher span values can result in a broad PSD and high polydispersity due to the breaking up of the larger agglomerated particles during the fluidization [23]. Consequently, the gum particle size is greatly influenced by the types of gum and sugar binder.

### 3.2. Flowability, Cohesiveness, and Porosity (ε)

The CI (7.67–18.5%) and HR (1.09–1.23) values of the GG and LBG agglomerates with various sugar binders (except for GG with sorbitol) were lower than those (19.0–19.7% for CI and 1.23–1.24 for HR) of the fine powders (Table 3), indicating that the agglomerates had better flowability and lower cohesiveness. Thus, in the FBA process, the sugar binder improved the flowability of the GG (except for GG with sorbitol) and LBG powders. However, the CMC agglomerates showed much higher CI and HR values than their fine powders, which was due to their relatively larger and more irregular particle shapes compared to the GG and LBG agglomerates, as illustrated in the SEM micrographs. Among the sugar binders, the sorbitol-based agglomerates incurred the highest CI and HR values, which indicates that the flowability of the agglomerates could be strongly influenced by the type of sugar binder. In particular, the CMC agglomerates incurred a significant particle size increase because of the strong solid bridges between the initial gum particles in the presence of the sugar binder, resulting in larger and more fibrous agglomeration than in the other agglomerates. The CI (GG: 12.0–14.9%; LBG: 7.67–10.0%) and HR (GG: 1.14–1.19; LBG: 1.09–1.11) values of the GG and LBG agglomerates with glucose, sucrose, and lactose were lower than the CI (GG: 21.7%; LBG: 18.5%) and HR (GG: 1.28; LBG: 1.23) values of the agglomerates with sorbitol. Thus, the GG and LBG agglomerates with sugar binders other than sorbitol exhibited very good flowability (CI < 15) and good cohesiveness (HR < 1.2). However, this was not the case for the CMC agglomerates with only fair (20 < CI <35) or bad flowability (35 < CI < 45) and high cohesiveness (HR > 1.4). For the CMC agglomerates, the higher CI and HR values can be attributed to lower ρ_tapped_ and ρ_bulked_ values due to the larger and more irregular particle sizes compared to the GG and LBG agglomerates. These results indicate that in the FBA process, the sugar binder considerably influenced the particle size and flow characteristics of the CMC agglomerates.

Porosity (ε) is very vital to the internal microstructure of agglomerated powders. The ε values of the agglomerates were much higher than those of the fine gum powders (Table 3), which can be attributed to the particle-clustering phenomenon due to FBA and the binder, resulting in the formation of large globular clusters [24]. An agglomerate with high porosity has an open and irregular structure with individual particles occasionally “sticking out” of the main agglomerate structure [13]. In the present study, the CMC agglomerates had higher ε values than the GG and LBG agglomerates, thus the large cluster formations of the CMC particles with the sugar binders were due to the good compatibility between them. In addition, the highest ε values of GG, LBG, and CMC agglomerates with different sugar binders were with glucose, sucrose, and sorbitol, respectively. These results indicate that the ε levels of the agglomerates were strongly affected by both the gum and sugar types due to their different intragranular porosities. The same effect has been observed in other studies on XG agglomerated with sugar binders [5] and galactomannans agglomerated with dextrin [4].

### 3.3. Powder Dispersibility

**Figure 2:**
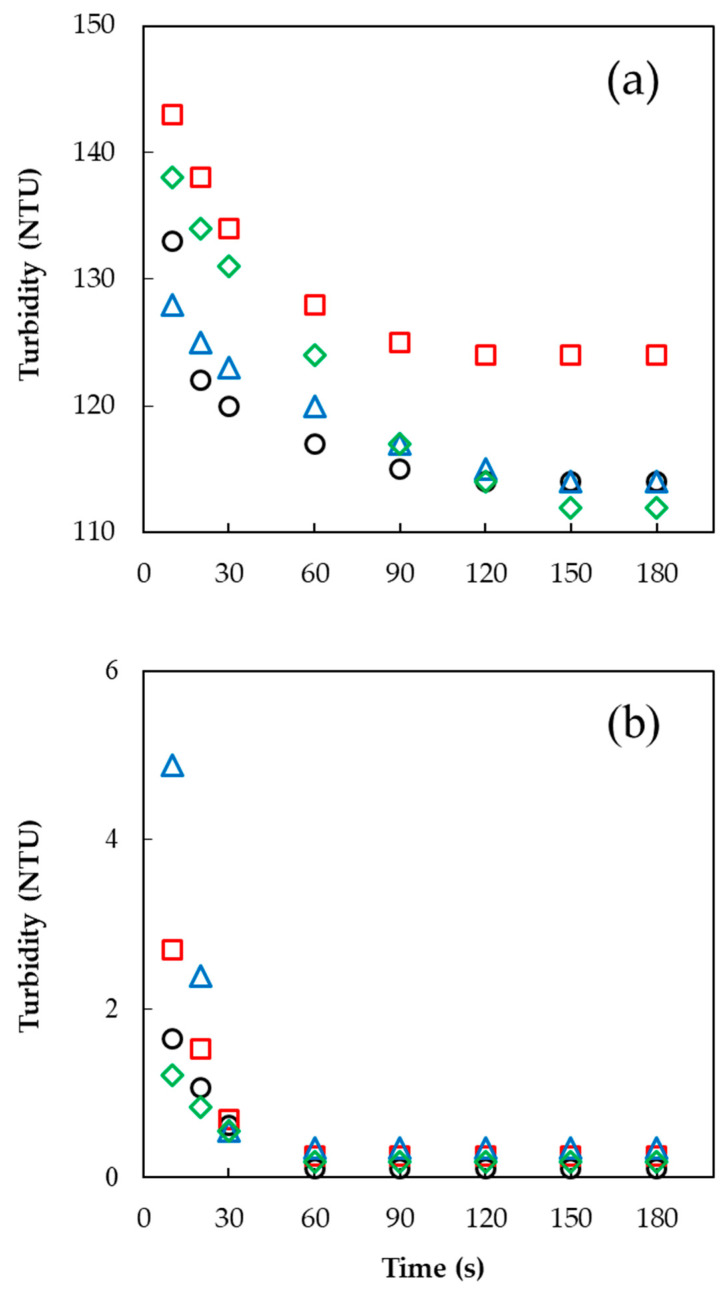
Turbidity value (NTU) of (**a**) GG and (**b**) CMC agglomerates with different sugar binders: (○) glucose, (□) sucrose, (△) lactose, and (◇) sorbitol.

**Figure 3:**
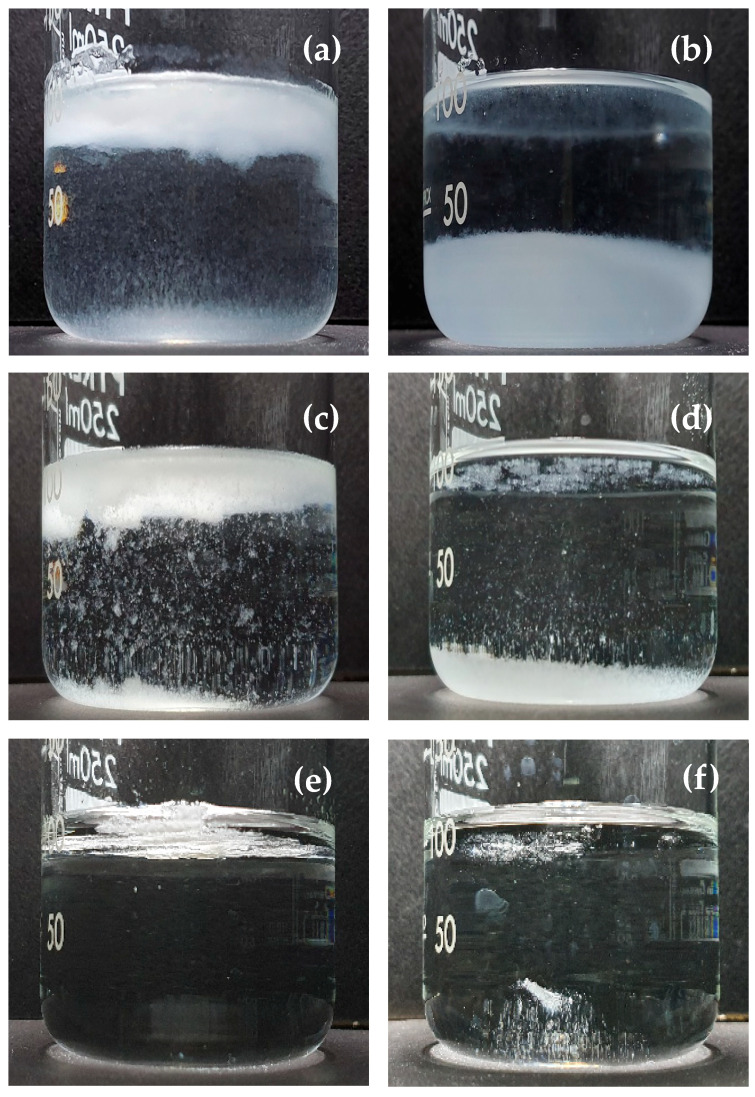
Water dispersibility of original fine gum powders (**a**–**e**) and gum agglomerates with sorbitol binder (**b**–**f**) in distilled water: (**a**,**b**) GG, (**c**,**d**) LBG, and (**e**,**f**) CMC.

This is indirectly observed via turbidimetry as a measure of the suspended or non-dissolved particles in a solution [25]. The turbidity values are obtained by measuring the loss of intensity of transmitted light due to the scattering effect of particles suspended in a transparent solution [26]. Gaiani et al. [27] identified the wetting, swelling, dispersion, and dissolution stages by analyzing turbidity profiles, and they found that there was a rate-limiting stage depending on the type of sample. Figure 2 shows the turbidity values of the GG and CMC agglomerates with sugar binders and their different dispersibility behaviors. Turbidity value as a function of stirring time was measured to investigate the effect of the sugar binder on the dispersibility of the GG and CMC agglomerates by observing lump formation and break-up while stirring the samples. There was a peak turbidity value followed by an abrupt decrease at around 30 s for CMC (90 s for GG) that reached a constant value at around 60 s for CMC (120 s for GG). The peak value of turbidity could be due to lump formation resulting from the interaction between the particles in water [25], whereas the abrupt decrease in turbidity could be due to the disintegration of lumps due to the stirring until a constant value is reached [28]. During stirring (10–180 s), the turbidity values (4.88–0.11 NTU) of the CMC agglomerates with sugar binders were much lower than those (143–112 NTU) of the GG agglomerates, while the solution remained almost transparent during the dispersal of the CMC agglomerates (Figure 2). The low turbidity values of CMC agglomerates during stirring meant that they were easy to disperse and dissolve without forming clusters. This can be attributed to the larger particle size of CMC agglomerates with minimal lump formation. According to Gaiani et al. [27], wetting, swelling, dispersion, and dissolution stages during the dispersal can be observed in the turbidity profile of a powder, and there is a rate-limiting stage depending on the type of product. In the current study, the GG agglomerates only reached the dispersion stage because they attained high turbidity values even after 180 s of stirring time. In contrast, the FBA process improved the dispersibility and dissolution of CMC without particle clustering. Different dispersion behavior between the fine gum powder and agglomerate was also observed after adding the gum powder to water in a beaker, as shown in Figure 3.

The agglomerate formation resulted in sunken and suspended particles without lumps floating on the water surface except for the CMC agglomerates, which produced transparent solutions without sunken and suspended particles. In general, the GG agglomerates with different sugar binders attained peak turbidity values that decreased in the order of sucrose (143 NTU) > sorbitol (138 NTU) > glucose (133 NTU) > lactose (128 NTU) (Figure 2), showing that at the beginning of dispersion, using lactose incurred less cloudiness than the other sugar binders. In contrast, the CMC decreased turbidity peak values in the order of lactose (4.88 NTU) > sucrose (2.70 NTU) > glucose (1.64 NTU) > sorbitol (1.21 NTU). Therefore, among the agglomerated gums, the fastest dispersing agglomerates reaching stabilization of turbidity were GG with lactose and CMC with sorbitol.

### 3.4. Morphology

**Figure 4:**
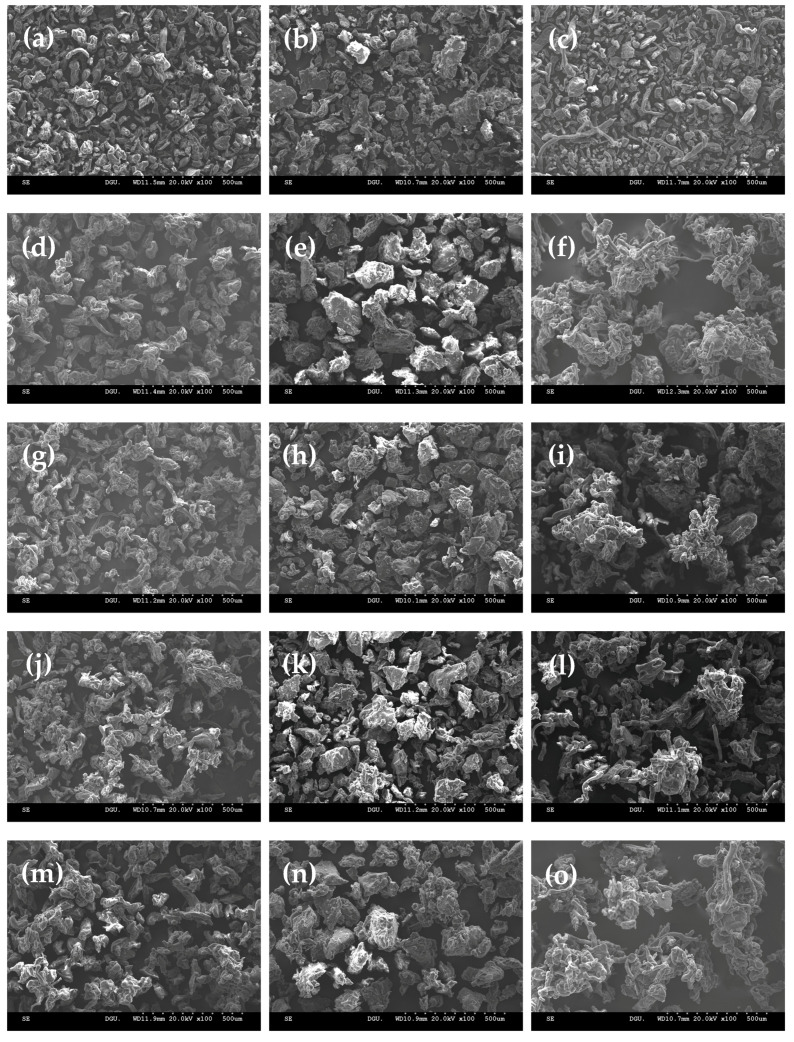
SEM micrograph for the fine gum powders (**a**–**c**) and gum agglomerates (GG, LBG, and CMC) with different sugar binders (**d**–**o**): (**d**–**f**) glucose, (**g**–**i**) sucrose, (**j**–**l**) lactose, and (**m**–**o**) sorbitol. Magnification 100×.

SEM was used to determine the structures of the original fine gum powder (Figure 4a–c) and the agglomerates with different sugar binders (Figure 4d–o). The particles in the fine gum powders displayed a small, compact, and smooth appearance, while the agglomerate showed large and irregular-shaped particles with wrinkled and porous structures due to the sticking of the smaller particles to the larger ones. It is well known that agglomerated particles can have different sizes, shapes, and structures [4,5]. The particle sizes of the agglomerate with sugar binders were much larger than those of the fine gum powder, indicating that the individual fine particles could stick together under the influence of the sugar binder. In general, among the agglomerates, the particle size increased in the following order: GG < LBG < CMC. Even though the particle size (106 μm) of the fine LBG powder was higher than those (56.7–82.5 μm) of the fine GG and CMC powders (Table 1), the addition of sugar binder solution incurred a larger and more irregular porous structure in the CMC agglomerate due to better intermolecular interaction between the thread-like particles. For the GG and LBG agglomerates with different sugars, those with glucose were less irregular, denser, and smoother than those with the other sugars that improved the powder flowability, as indicated by the lower CI (12.0% for GG and 7.67% for LBG) and HR (1.14 for GG and 1.09 for LBG) values of the GG and LBG agglomerates with glucose (Table 3), as mentioned previously. Overall, the addition of the various sugar binders resulted in significantly different morphologies in the resulting agglomerates, demonstrating that the size and shape of the latter were considerably influenced by the type of sugar binder.

### 3.5. Colorimetric Analysis

In general, color is crucial for the sensory quality of a powder product. The agglomeration of gum powder modified the color parameters (L *, C *, and ΔE *) observed with a colorimeter (Table 4), even though the visual appearance showed a slight color change. The L * values of the LBG and CMC agglomerates with sorbitol were lower than the respective fine powders, whereas those of GG were higher. The L * values of all of the agglomerates (except for GG with glucose or sorbitol) were significantly lower than those of the respective fine gum powders, indicating that they were slightly darker in color due to hot-air drying during the FBA process. There were no noticeable differences in L * value among the LBG and CMC agglomerates, irrespective of the type of sugar. However, the CMC powders showed great differences in L * values between the agglomerates with sugars (L * = 83.7–84.7) and the fine gum powder (L * = 88.3). This indicates that the lightness of the CMC powder was greatly influenced by the agglomeration with sugar binder. GG agglomerated with lactose and LBG and CMC agglomerated with sorbitol showed the lowest L * values because of their larger particle sizes compared to the other agglomerates (Table 1), indicating that the L * value was affected by particle size. According to Sakhare et al. [29], a small particle size results in a great increase in whiteness.

Regarding C *, which is related to color intensity, the agglomerates showed much higher C * values (10.9–14.6) compared to the fine gum powders (10.4–13.0), and the change in color of the agglomerates was more influenced by the type of gum than the type of sugar. The ΔE * values (3.69–4.68) of the CMC agglomerates were much higher than those (1.42–2.29) of the others. In addition, except for GG, there was not much difference between the ΔE * values of the agglomerates according to the type of sugar, indicating that the ΔE * was more influenced by the type of gum than the type of sugar binder.

## 4. Conclusions

The physical properties of three agglomerates prepared with fine gum powders and four sugar binders using a fluidized-bed granulator were investigated in this study. In general, among the agglomerates, the particle size increased in the following order: GG < LBG < CMC, indicating that for CMC, the addition of a sugar binder solution incurred larger and more irregular porous structures due to better intermolecular interaction between the threadlike particles. In addition, the higher particle enlargement in the CMC agglomerates was due to the stronger solid bridge formations connecting the primary particles with the sugar binder. The flow characteristics of the agglomerates were strongly influenced by the size and shape of agglomerated particles. The GG and LBG agglomerates showed better flowability and lower cohesiveness due to their relatively smaller and less irregular particle shapes compared to CMC, as can be seen in the SEM images. The CMC agglomerates with sugar binders showed higher porosity values due to good compatibility between the CMC particles and sugar binders. The turbidity values of the CMC agglomerates were much lower than those of GG, indicating that agglomeration improved the dispersibility and dissolution of CMC without particle clustering. The agglomeration process of the gum powders incurred color modification, with the changes in ΔE * being significantly affected by the gum type. Overall, the sugar binder addition in the FBA process was beneficial and improved the physical properties of the fine gum powders by greatly influencing the structure of the primary particles. Specific knowledge of the physical characteristics of a gum agglomerate would be helpful for selecting the appropriate sugar binder solution in the FBA process.

## Figures and Tables

**Table 1:** Particle size distribution of fine gum powders and gum agglomerates (GG, LBG, and CMC) with different sugar binders.

Gum Powder Type	Binder Type	D_10_ (μm)	D_50_ (μm)	D_90_ (μm)	Span
Fine GG		25.0 ± 0.1 ^e^	56.7 ± 0.1 ^e^	108.9 ± 0.5 ^d^	1.48 ± 0.01 ^d^
GG agglomerate	Glucose	49.4 ± 0.1 ^c^	93.9 ± 0.1 ^c^ (39)	179.4 ± 0.3 ^c^	1.39 ± 2·10^−3 e^
	Sucrose	41.6 ± 0.1 ^d^	89.5 ± 0.4 ^d^ (58)	179.4 ± 1.1 ^c^	1.54 ± 5·10^−3 c^
	Lactose	53.7 ± 0.4 ^b^	169.8 ± 0.4 ^a^ (200)	445.7 ± 1.4 ^a^	2.31 ± 0.01 ^a^
	Sorbitol	56.3 ± 0.5 ^a^	124.4 ± 0.4 ^b^ (117)	293.0 ± 1.2 ^b^	1.90 ± 0.01 ^b^
Fine LBG		33.1 ± 0.1 ^e^	106.3 ± 0.1 ^d^	203.1 ± 0.1 ^e^	1.60 ± 1·10^−3 a^
LBG agglomerate	Glucose	78.7 ± 0.3 ^b^	143.3 ± 0.4 ^b^ (35)	238.1 ± 0.1 ^c^	1.11 ± 3·10^−3 d^
	Sucrose	68.5 ± 0.1 ^d^	135.0 ± 0.2 ^c^ (27)	233.2 ± 0.2 ^d^	1.22 ± 2·10^−3 c^
	Lactose	73.4 ± 0.3 ^c^	142.0 ± 0.7 ^b^ (34)	246.9 ± 0.9 ^b^	1.22 ± 0.01 ^c^
	Sorbitol	86.4 ± 0.3 ^a^	170.5 ± 0.3 ^a^ (61)	310.8 ± 0.9 ^a^	1.32 ± 4·10^−3 b^
Fine CMC		26.6 ± 0.1 ^d^	82.5 ± 0.1 ^d^	222.2 ± 0.6 ^d^	2.37 ± 5·10^−3 a^
CMC agglomerate	Glucose	99.7 ± 0.5 ^a^	217.5 ± 1.2 ^a^ (163)	400.4 ± 0.1 ^b^	1.38 ± 0.01 ^d^
	Sucrose	87.3 ± 0.2 ^c^	215.7 ± 0.6 ^b^ (159)	408.4 ± 1.2 ^a^	1.50 ± 0.01 ^b^
	Lactose	90.2 ± 0.5 ^b^	206.6 ± 1.5 ^c^ (151)	391.6 ± 1.9 ^c^	1.46 ± 4·10^−3 c^
	Sorbitol	99.0 ± 0.3 ^a^	218.3 ± 0.7 ^a^ (164)	400.2 ± 0.9 ^b^	1.38 ± 5·10^−3 d^

D_10_, D_50_, and D_90_ are values of the particle diameter at 10%, 50%, and 90% in the cumulative size distribution, respectively. Values are means of three measurements ± SD. Means with different lowercase letters (a–e) within each column are significantly different (*p* < 0.05). Percentage increase in D_50_ is between non-agglomerated and agglomerated gum powders.

**Table 2:** Capillary viscosity (η_c_) values for sugar binder solutions at 10% w/w.

Binder Type	η_c_ (mPa·s)
Glucose	1.161 ± 0.001 ^c^
Sucrose	1.182 ± 1·10^−4 b^
Lactose	1.186 ± 1·10^−4 a^
Sorbitol	1.154 ± 0.001 ^d^

Values are means of triplicate measurements ± SD. Means with different lowercase letters (a–d) within each column are significantly different (*p* < 0.05).

**Table 3:** Physical properties of fine gum powders and gum agglomerates (GG, LBG, and CMC) with different sugar binders.

Gum Powder Type	Binder Type	ρ_bulk_ (g/cm^3^)	ρ_tapped_ (g/cm^3^)	Porosity (%)	CI (%)	HR (-)
Fine GG		0.58 ± 1·10^−3 a^	0.71 ± 0.01 ^a^	57.2 ± 0.1 ^d^	19.0 ± 1·10^−3 b^	1.23 ± 1·10^−3 b^
GG agglomerate	Glucose	0.43 ± 1·10^−3 b^	0.49 ± 1·10^−3 b^	77.0 ± 2.9 ^a^	12.0 ± 1·10^−3 e^	1.14 ± 1·10^−3 e^
	Sucrose	0.40 ± 0.01 ^c^	0.47 ± 1·10^−3 c^	63.7 ± 2.7 ^c^	14.9 ± 0.4 ^c^	1.19 ± 0.01 ^c^
	Lactose	0.37 ± 1·10^−3 d^	0.43 ± 0.01 ^d^	65.4 ± 1·10^−3 c^	14.0 ± 0.4 ^d^	1.17 ± 0.01 ^d^
	Sorbitol	0.31 ± 0.01 ^e^	0.39 ± 0.01 ^e^	72.7 ± 1·10^−3 b^	21.7 ± 0.6 ^a^	1.28 ± 1·10^−3 a^
Fine LBG		0.58 ± 0.01 ^a^	0.73 ± 0.01 ^a^	71.0 ± 1·10^−3 e^	19.7 ± 0.6 ^a^	1.24 ± 0.01 ^a^
LBG agglomerate	Glucose	0.50 ± 0.01 ^d^	0.59 ± 0.01 ^c^	88.3 ± 1·10^−3 b^	7.7 ± 0.6 ^c^	1.09 ± 0.01 ^d^
	Sucrose	0.52 ± 1·10^−3 c^	0.57 ± 1·10^−3 d^	88.6 ± 1·10^−3 a^	9.5 ± 0.7 ^b^	1.11 ± 0.01 ^c^
	Lactose	0.54 ± 1·10^−3 b^	0.60 ± 1·10^−3 b^	82.0 ± 1·10^−3 d^	10.0 ± 1·10^−3 b^	1.11 ± 1·10^−3 c^
	Sorbitol	0.42 ± 1·10^−3 e^	0.52 ± 0.01 ^e^	84.7 ± 1·10^−3 c^	18.5 ± 0.7 ^a^	1.23 ± 0.01 ^b^
Fine CMC		0.57 ± 1·10^−3 a^	0.77 ± 1·10^−3 a^	48.4 ± 1·10^−3 c^	26.7 ± 0.6 ^d^	1.36 ± 0.01 ^d^
CMC agglomerate	Glucose	0.18 ± 1·10^−3 c^	0.28 ± 1·10^−3 c^	90.8 ± 1·10^−3 a^	34.0 ± 1·10^−3 c^	1.52 ± 1·10^−3 c^
	Sucrose	0.21 ± 1·10^−3 b^	0.32 ± 1·10^−3 b^	78.7 ± 1·10^−3 b^	35.0 ± 1·10^−3 b^	1.54 ± 1·10^−3 b^
	Lactose	0.21 ± 1·10^−3 b^	0.32 ± 1·10^−3 b^	78.8 ± 1·10^−3 b^	34.0 ± 1·10^−3 c^	1.52 ± 1·10^−3 c^
	Sorbitol	0.16 ± 1·10^−3 d^	0.26 ± 1·10^−3 d^	91.2 ± 1·10^−3 a^	39.0 ± 1·10^−3 a^	1.64 ± 1·10^−3 a^

Values are means of triplicate measurements ± SD. Means with different lowercase letters (a–e) within each column are significantly different (*p* < 0.05).

**Table 4:** Color parameters of fine gum powders and gum agglomerates (GG, LBG, and CMC) with different sugar binders.

Sample	Binder Type	L *	C *	ΔE *
Fine GG		81.8 ± 0.1 ^c^	12.9 ± 1·10^−3 c^	-
GG agglomerate	Glucose	82.8 ± 1·10^−3 a^	14.2 ± 0.1 ^a^	1.75 ± 0.03 ^b^
	Sucrose	79.9 ± 0.1 ^d^	13.9 ± 1·10^−3 b^	2.20 ± 0.06 ^a^
	Lactose	79.9 ± 0.1 ^d^	14.1 ± 1·10^−3 a^	2.29 ± 0.06 ^a^
	Sorbitol	82.4 ± 1·10^−3 b^	13.9 ± 1·10^−3 b^	1.42 ± 0.06 ^c^
Fine LBG		82.9 ± 0.1 ^a^	13.0 ± 0.1 ^c^	-
LBG agglomerate	Glucose	82.5 ± 0.1 ^b^	14.6 ± 1·10^−3 a^	1.76 ± 0.19 ^a^
	Sucrose	82.7 ± 0.1 ^ab^	14.5 ± 1·10^−3 ab^	1.64 ± 0.14 ^a^
	Lactose	82.8 ± 0.1 ^ab^	14.3 ± 1·10^−3 b^	1.54 ± 0.13 ^a^
	Sorbitol	82.1 ± 0.1 ^c^	14.4 ± 0.1 ^ab^	1.72 ± 0.36 ^a^
Fine CMC		88.3 ± 1·10^−3 a^	10.4 ± 1·10^−3 d^	-
CMC agglomerate	Glucose	84.5 ± 0.2 ^b^	11.1 ± 1·10^−3 b^	3.92 ± 0.21 ^b^
	Sucrose	84.7 ± 0.2 ^b^	10.9 ± 1·10^−3 c^	3.69 ± 0.21 ^b^
	Lactose	84.6 ± 1·10^−3^ ^b^	11.3 ± 1·10^−3 a^	3.81 ± 1·10^−3 b^
	Sorbitol	83.7 ± 1·10^−3^ ^c^	11.3 ± 0.1 ^a^	4.68 ± 0.01 ^a^

Values are means of triplicate measurements ± SD. Means with different lowercase letters (a–d) within each column are significantly different (*p* < 0.05).

## Data Availability

All the results given in the manuscript can be requested from the corresponding author, who will provide them.
